# Timed Up & Go as a measure for longitudinal change in mobility after stroke – Postural Stroke Study in Gothenburg (POSTGOT)

**DOI:** 10.1186/1743-0003-11-83

**Published:** 2014-05-09

**Authors:** Carina U Persson, Anna Danielsson, Katharina S Sunnerhagen, Anna Grimby-Ekman, Per-Olof Hansson

**Affiliations:** 1 Institute of Neuroscience and Physiology, Department of Clinical Neuroscience and Rehabilitation, Rehabilitation Medicine, Sahlgrenska Academy, University of Gothenburg, Per Dubbsgatan 14, 413 45 Göteborg, Sweden; 2 Occupational and Environmental Medicine, Sahlgrenska Academy, University of Gothenburg, Göteborg, Sweden; 3 Institute of Medicine, Sahlgrenska Academy, University of Gothenburg, Göteborg, Sweden

**Keywords:** Mobility limitation, Clinometric, Recovery of function, Outcome, Responsiveness

## Abstract

**Background:**

A frequently used clinical test to assess mobility after stroke is the Timed Up & Go. Knowledge regarding whether or not the Timed Up & Go is able to detect change over time in patients with stroke, whether improvements in mobility exist after the first three months and whether or not longitudinal change in mobility after stroke depend on the patients’ age, is limited or unclear. The objectives were to investigate the distribution-based responsiveness of the Timed Up & Go (TUG) during the first three months after a first event of stroke, to measure the longitudinal change in TUG time during the first year after stroke and to establish whether recovery in TUG time differs between different age groups.

**Methods:**

Ninety-one patients with first-ever stroke were assessed using the Timed Up & Go at the 1st week and at 3, 6 and 12 months after stroke. The non-parametric sign-test, the parametric t-test and a mixed model approach to linear regression for repeated measurements (Proc mixed) were used for the statistical analyses.

**Results:**

The median TUG time was reduced from 17 to 12 seconds (p < 0.001) between the 1st week and 3 months. No further improvement was seen between 3 and 12 months after stroke. In a mixed model approach to linear regression, there was a significant age difference. Patients at age 80 and above tended to deteriorate in terms of TUG time between 3 and 12 months after stroke, while patients < 80 years did not (p = 0.011 for the interaction between age group and time).

**Conclusion:**

The Timed Up & Go demonstrates ability to detect change in mobility over time in patients with stroke. A significant improvement in TUG time from the 1st week to 3 months after stroke was found, as expected, but thereafter no statistically significant change was detected. After 3 months, patients ≥80 years tended to deteriorate in terms of TUG time, while the younger patients did not.

## Background

Mobility limitations are commonly observed in people after a stroke [1,2]. In stroke rehabilitation, measures of change and improvement are essential both in establishing natural history and in evaluating rehabilitation interventions, why the clinometric characteristics of outcome measures should be established. A frequently used clinical test to assess functional mobility is the Timed Up & Go (TUG) [3]. The TUG has shown varying degrees of inter- and intra-rater reliability in patients with chronic stroke, as well as in patients in geriatric day care [3-7]. Furthermore, the TUG has been shown to be valid and to identify the risk of falling in community-dwelling older adults [8] as well as in patients with stroke [2,9]. For the latter study [2], the risk of falling was identified as the inability to perform the TUG. One aspect of validity is responsiveness, defined as “the ability of an instrument to detect change over time in the construct being measured” [10].

To our knowledge, only two prior studies have addressed the responsiveness of the TUG in patients with stroke [11,12]. Both of these studies were small (n = 50 and 44, respectively) [11,12] and in one of the studies the most disable patients were excluded from the analysis [11]. Consequently, further studies are needed. There is no gold standard when it comes to assessing responsiveness, but it is essential that clinical status is expected to change [13-17]. Responsiveness relying on statistical properties is described as “distribution-based” [16], which is a way to express the observed change in a standardized metric within a sample. Several studies confirm that neurological recovery mainly takes place early after stroke [18-23]. Whether improvements in mobility exist after the first three months and whether or not longitudinal change in mobility after stroke depend on the patients’ age, is still unclear [20,23-25].

The aim of the present study was to investigate the distribution-based responsiveness of the TUG in patients with a first event of stroke during the first 3 months after stroke, assuming an improvement in functional mobility during this period [24,26]. Additional objectives were to establish the longitudinal change in functional mobility during the 1st year after stroke, and to study whether recovery in mobility after stroke differs in different age groups. Functional mobility is hereafter referred to as mobility or TUG time.

## Methods

### Subjects

This study is a follow-up part of the Postural Stroke Study in Gothenburg (POSTGOT) [2]. The POSTGOT consists of 116 consecutive patients, admitted to the stroke unit at Sahlgrenska University Hospital/Östra, Gothenburg, Sweden, after a 1st event of stroke. The stroke diagnosis was defined according to World Health Organization criteria [27]. The exclusion criteria were co-morbidities such as leg amputation, a diagnosis of dementia or severe psychiatric diseases that could interfere with mobility or the ability to cooperate during the assessments. Ischemic stroke events were classified according to the Trial of Org 10172 in Acute Stroke Treatment (TOAST) criteria [28]. The Regional Ethics Committee of Gothenburg approved the study and informed written consent was obtained from the patient or next of kin prior to study entry, according to the Declaration of Helsinki.

### Outcome Measures and Procedures

According to clinical routine, the patients were assessed using the Modified Motor Assessment Scale-95 (M-MAS UAS-95) and the Berg Balance Scale during the 1st week after stroke.

The patients’ mobility was investigated using the TUG on 4 different occasions. The 1st assessment was performed during the 1st week after stroke onset, between days 4 and 7, median day 5. All the TUG assessments were performed in the corridor on the ward, by a physiotherapist not involved in the patient’s rehabilitation. The TUG was performed as follows: the patients were asked to stand up from a standardized armchair, walk 3 meters (marked by a tape), turn, walk back to the chair and sit down, as quickly and as safely as possible while the time taken to complete the test was recorded and rounded to whole seconds. In cases where the patient needed a walking aid, his/her private walking aid was used. No physical assistance was accepted.

The patients were then followed-up using TUG with assessments at 3, 6 and 12 months after stroke. In addition, at the same times, the patients were also assessed using the M-MAS UAS-95.

All patients were invited to each follow-up assessment, irrespectively of whether or not they had previously participated. For the follow-up examinations, a time window of ±14 days was allowed. Data on recurrent stroke, after the 1st event of stroke, were collected from the medical records. If the patient had suffered a recurrent stroke, the results from the follow-ups after the 2nd stroke were excluded from further analysis.

### Data analysis

For all statistical analyses, the SAS version 9.3 (SAS Institute, Cary, NC) was used. All tests were two-tailed. P-values of less than 0.05 were considered statistically significant. The non-parametric sign test was used to investigate whether patients had “improved” or “deteriorated” between two time points (from 1st week to 3 months, 3 to 6 months and 6 to 12 months). Patients unable to perform TUG were included using a surrogate time. The surrogate time was slower than the slowest time of the entire study period and the same for all the assessment occasions. “Improved” was defined as reduced TUG time or going from *unable* to *able* to perform TUG. “Deteriorated” was defined as increased TUG time or going from *able* to *unable* to perform TUG. In an additional analysis, the parametric paired t-test was performed including only those patients who were able to perform the TUG at 1st week and 3 months after stroke.

The longitudinal change in TUG time, from 3 to 12 months after stroke, was further analyzed in a mixed model approach to linear regression for repeated measurements (Proc mixed, SAS procedure). In this regression model, time after stroke, age group and the interaction of these two factors were used as fixed explanatory variables. The age limits; 45–64 years, 65–79 years and 80 years of age and above, were based on PubMed’s division for middle aged (45–64 years), aged (65+ years) and 80 year and older (80+ years). In addition a random intercept was included. The random intercept handles the dependence within individuals present in data when individuals are measured repeatedly over time. Analyses were also carried out with all data, from the 1st week to 12 months (including data from the responsiveness analysis).

## Results

**Figure 1:**
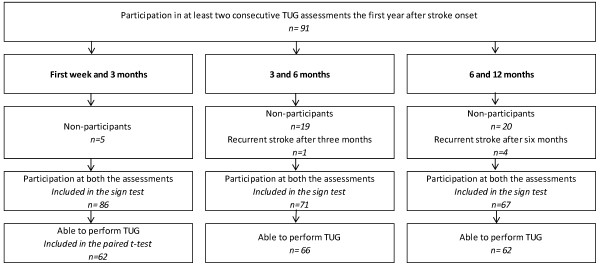
Flow chart showing patients included and excluded in the follow-up analyses at first week and 3 months, at 3 and 6 months and at 6 and 12 months.

The population of the present study comprised the 91 patients who participated in at least two consecutive TUG assessments during the first year after stroke onset. Table 1 presents the patients’ characteristics and the median scores from other clinical scales at baseline. The patients had a median length of stay of 14 days (range: 4–79 days) at the stroke unit, where physiotherapy and occupational therapy were offered 5 days a week. Of the 91 patients in the study, 75 patients (82%) were discharged to their own homes, 13 to nursing homes, two to a geriatric rehabilitation clinic and one patient to a rehabilitation medicine clinic. Figure 1 shows a flow-chart diagram of patients included and excluded in the follow-up analyses. Table 2 illustrates the different categorical changes, based on TUG time, between two consecutive assessments in the three analyses: at 1st week and 3 months, at 3 and 6 months and at 6 and 12 months after stroke. Table 3 displays the Timed Up & Go time for the patients able to perform the test for each of the 4 different time points for the assessments.

**Table 1:** Baseline characteristics and median values for the Berg Balance Scale (BBS) and the Modified Motor Assessment Scale (M-MAS UAS-95) one to seven days after stroke onset

**Characteristics, n = 91**	**n (%) or median (range)**
Female	38 (42)
Age, years	72.6 (47–94)
Stroke classifications (TOAST)	
Large vessel disease	23 (25)
Small vessel disease	25 (27)
Cardioembolic stroke	19 (21)
Cryptogenic stroke	15 (17)
Hemorrhagic stroke	9 (10)
Right-side lesion	43 (47)
Left-side lesion	48 (53)
Hypertension	58 (36)
Diabetes mellitus	23 (25)
Berg Balance Scale n = 88	41 (0–56)
M-MAS UAS-95 n = 80	49 (12–55)

TOAST; Trial of Org 10172 in Acute Stroke Treatment, M-MAS UAS-95; Modified Motor Assessment Scale Uppsala Akademiska Hospital -95.

**Table 2:** Different outcomes in number of categorical changes based on Timed Up & Go (TUG) time between two consecutive assessments in the three analyses, from first week to three months, from three to six and from six to twelve months after stroke

		**Months after stroke**		
**Outcomes, n**		**0 to 3**	**3 to 6**	**6 to 12**
		**n = 86**	**n = 71**	**n = 67**
Improved	Unable to able to perform TUG	15	1	0
	Reduced TUG time	51	28	24
Unchanged	Unchanged TUG time	13	23	15
Deteriorated	Increased TUG time	7	19	27
	Able to unable to perform TUG	0	0	1

**Table 3:** Timed up & go time, in seconds, for the patients able to perform the test for each of the four time points for assessment

	**First week**	**3 months**	**6 months**			**12 months**
	**n = 68**	**n = 77**	**n = 71**		**n = 70**	
Mean (SD)	17.0 (11.0)	14.5 (10.0)	14.2 (9.4)		14.7 (9.8)	
Median (IQR)	13.0 (10.6-18.0)	11.0 (10.0-16.3)	11.5 (10.0-16.0)		12.0 (9.0-17.0)	

Time specified after stroke onset. SD; Standard Deviation, IQR; Inter Quartile Range (i.e. the 25th and 75th percentile).

### 1st week to 3 months

A large proportion of the patients, 24 out of 86 (28%), were unable to perform the test at the first assessment, while 9 patients (10%) were unable to do this at the 3 months assessment (Table 2). Sixty-six patients (77%) improved and 7 (8%) patients deteriorated from 1st week to 3 months, p <0.001. The median improvement in TUG time, based on all 86 patients, was five seconds (from 17 to 12 seconds). When analyzing only the 62 patients who were able to perform the TUG both in the 1st week and at the 3-months assessment, the mean TUG time was reduced by 5.3 seconds (95% CI 2.9-7.6, p < 0.001), from 17.5 (95% CI 14.7-20.3) to 12.2 (95% CI 11.2-13.3) seconds. The median M-MAS UAS-95 score, for the patients who participated in both assessments, increased from 49 to 55 (p < 0.001).

### 3 to 6 months

One patient experienced a recurrent stroke after the 3-month follow-up and was therefore excluded. Consequently, the analyses are based on 71 patients. Five of these (7%) were unable to perform the test at 3 months while four patients (6%) were still unable to do this at 6 months. Between 3 and 6 months, the median TUG time went from 11 to 12 seconds. The change was non-significant. The corresponding median M-MAS UAS-95 score went from 55 to 54 (non-significant).

### 6 to 12 months

Four of the 67 patients (6%) who participated in both assessments were unable to perform the test at 6 months while further one more, totally five patients (7%), were unable to perform the TUG at 12 months. Between 6 and 12 months, the median TUG value went from 11 to 12 seconds in TUG time. The change was non-significant. The corresponding median M-MAS UAS -95 score went from 54 to 55 (non-significant).

**Figure 2:**
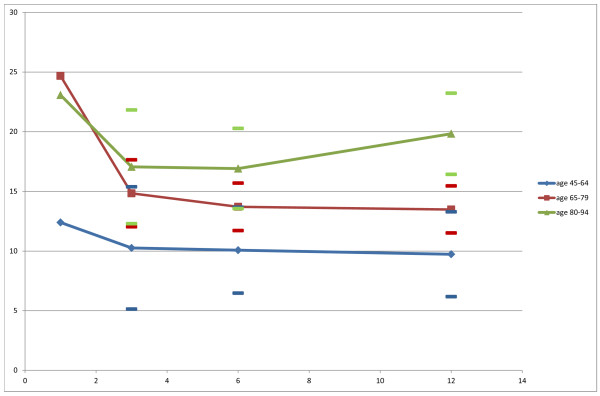
**The recovery in TUG time for each of the three age groups. **The x-axis represents time, in months, after stroke and the y-axis the Timed Up & Go time in seconds. Solid lines represent the model means (lsmeans) and dashes the associated 95% confidence intervals.

Figure 2 illustrates the results for the recovery in TUG time based on the regression analysis. The regression analysis was based on 211 observations of 81 patients who participated in any of the 3 follow-up assessments (n = 70 at 3 months, n = 74 at 6 months, n = 67 at 12 months) and who were able to perform the TUG. The analysis showed the following results; age group p = 0.018, time p = 0.085 and the interaction between age group and time p = 0.011. The patients at age 80 and older tended to deteriorate from 3 to 12 months, while younger patients did not. Rather, there was a small tendency towards improvement among patients 64 years of age and younger. Figure 2 also illustrates the TUG time, from the 1st week to 3 months for the different age groups, based on data from the responsiveness part.

## Discussion

Our findings demonstrate that the TUG is a responsive test for capturing improvements in mobility during the first three months after stroke. Responsiveness was established in relation to statistically significant change, referred to as distribution-based responsiveness [16]. Another method for responsiveness analysis is to perform test-retest reliability studies to estimate the measurement error. If the size of a change over time is greater than the measurement error, the test is considered responsive. Thus, that the current mean reduction in TUG time of 5.3 seconds (95% CI 2.9-7.6) is larger than the previously published size of standard error of measurement (SEM), ranging from 1.14-1.78 seconds [4,6] provides further evidence that TUG is responsive to change. Noteworthy, still, is that the previously presented SEM values are based on rather small populations of patients with chronic stroke, which means that any comparisons should/must be made with some caution.

Another aspect of responsiveness concerns whether or not it is of clinical importance [29]. The fact that there is a statistical significant change in TUG time and that it shows distribution-based responsiveness does not necessary mean that this improvement is important for the patient. However, previous studies have shown that patients with a poor performance in TUG had a significant higher risk of falling after stroke [2,9]. Patients with TUG time of ≥15 s were at higher risk of falling during 1-year follow-up after stroke (2). The highest risk of falling was found for patients unable to perform TUG. Therefore, a median change in TUG time from 17 to 12 s seems to be of clinical importance. Furthermore, the fact that 15 of 24 patients went from unable to able to perform the TUG from the 1st week to 3 months post stroke could also be interpreted as being of clinical importance. In addition, the parallel detection of patient improvement, in median M-MAS UAS-95 score from first week to 3 months after stroke, validates that the direction of the TUG time change was expected. This strengthens the conclusion that the TUG is a relevant measure in clinical practice.

Our findings from the mixed model approach to linear regression supply information about the longitudinal change in TUG time after stroke for different age groups. The central issue is the declining recovery in mobility that was found from 3 to 12 months after stroke for the patients aged 80 and over. One year after stroke, these patients were almost back to the same level of TUG time as at the 1st week after stroke onset. Discussed in the context of clinical implication, the result justifies the use of the TUG in the rehabilitation following stroke.

Salbach *et al.*[11] studied the responsiveness of the TUG, based on assessments within 8 days and 4 weeks after stroke, among 50 ambulatory patients with a first-ever stroke with a mean age of 68 years. In that study, 20% of the patients were unable to perform the test at the first assessment and 6% at the second assessment. In addition, in their analysis the most disabled patients, i.e. those who were unable to walk 14 meters, were excluded. For the patients able to perform the TUG, the standardized response mean (SRM) was 0.73 (a moderate effect size). The improvement during the first month reported (mean TUG time from 22.2 ± 17.2 to 12.6 ± 5.5 seconds, p >0.01) for those patients who were able to perform the test), is slightly larger than the one that was found during the first three months after stroke, based on all patients, including those unable to perform the TUG. Furthermore, Knorr *et al.*[12] studied the sensitivity to change in TUG time between 3 and 8 months after stroke among 44 patients with a mean age of 63 years. They found a statistically significant improvement in mean TUG time, from 16.7 ± 17.1 seconds to 13.7 ± 16.0 seconds, p < 0.010, during this time, with a SRM of 0.34 (a small effect size). This result is different from the current study, where no clear over-all improvement from 3 to 12 months could be found. Though, to assess responsiveness on the basis of effect size has been criticized and is suggested to supply only partial evidence for responsiveness [30].

Moreover, the time and length of follow-up could affect the results. In a study with a long follow-up, the patients are more likely to suffer from other impaired health conditions, especially if they are of a high age. Even if we excluded patients who were hospitalized for a recurrent stroke during follow-up, additional factors such as activity level, as well as physical therapy received, might explain the varying results between the present and the previously referred studies. Only 36% of the patients were assessed as requiring further rehabilitation at discharge from the stroke unit. The intensity of this further rehabilitation is unknown. Clearly, it would be of great clinical interest to study the effect of intense training among stroke patients of different ages in a clinical randomized trial.

There are some limitations that restrict the generalization of our results. Only patients with a first ever clinical stroke were studied and the outcome cannot with certainty be transferred to patients with recurrent stroke. In addition, of the initial 116 patients in the POSTGOT study, 22% of the patients did not participate in the follow-up assessments. There is a risk of selection bias, as the most disabled patients might be less likely to participate in the follow-up. Furthermore, it is not possible to differentiate between improvements due to spontaneous neurological recovery or improvements caused by rehabilitation or living environment. Change in TUG time may also depend on impairment and recovery of muscle strength in the lower extremity. Previous research has shown that there is a correlation between the TUG and the peak plantar flexion torque [5]. TUG specific demands as standing up, standing and sitting down, seem to be functionally addressed to falls after stroke due to reduced force generation and greater postural sway [31,32]. Moreover, in persons with stroke, incidence of falls related to 180 degrees turn around in TUG may be due to deficits in cognitive processes [33]. It would also have been interesting to have a closer follow-up during the first three months to determine when the largest improvement in mobility occurred. Also, the fact that no comparisons can be made with some global scale is a limiting factor. Finally, the initial assessment was performed an average of 5 days after stroke. Due to practical reasons, the patients were not assessed before day 4–7. Thus, the present study is not able to answer the question whether TUG is appropriate as a clinical measure 1–3 days post stroke. However, motor recovery may already take place during the first days after stroke, which can explain why some patients may already have recovered before the first assessment.

On the other hand, strengths of the present study are that the stroke patients were unselected and investigated in the acute phase and followed repeatedly for one year. Moreover, the study population was somewhat larger compared with previous studies. Our findings provide increased knowledge relating to the interpretation of the TUG in clinical stroke rehabilitation. The results also contribute to knowledge regarding recovery in mobility after stroke, in general and for different age groups. To our knowledge, no previous studies have described the effect of age on recovery of mobility, expressed as TUG time, after stroke.

## Conclusion

In conclusion, our results indicate that the Timed Up & Go demonstrates ability to detect change in mobility over time in patients with stroke. Thus, the result justifies the use of TUG in stroke rehabilitation. As expected, a statistically significant improvement in TUG time from the 1st week to 3 months after stroke was found, but thereafter no statistically significant change could be detected. The recovery pattern of mobility differed between different age groups. Patients 80 years or older tended to deteriorate in mobility between 3 to 12 months after stroke, while the younger patients did not.

## Abbreviations

BBS: Berg Balance Scale; M-MAS UAS-95: Modified Motor Assessment Scale Uppsala Akademiska Sjukhus-95; POSTGOT: Postural Stroke Study in Gothenburg; SEM: Standard Error of Measurement; TOAST: Trial of Org 10172 in acute stroke treatment; TUG: Timed Up & Go.

## Competing interests

The authors declare that they have no competing interest.

## Authors’ contributions

All authors have made substantial contributions to the manuscript. KSS was primary responsible, and CUP and POH were involved, in the conception and design of the study. CUP was primary responsible in the collection of data. CUP and POH were primary responsible, but all authors have been involved, in the interpretation of data. AGE and CUP was primary responsible for statistical analyses, AGE as statistical expertise. CUP and POH were primary responsible in drafting the manuscript. AD, KSS and AGE have been involved in drafting the manuscript. All authors have read and approved the final manuscript.
